# Supplementary material for: Biosecurity and Lairage Time versus Pork Meat Quality Traits in a Farm–Abattoir Continuum
Source: Animals (Basel). 2022 Dec 1;12(23):3382. doi: 10.3390/ani12233382 (PMC9738693; doi:10.3390/ani12233382)
Supplement: Supplementary file 1 [file animals-12-03382-s001.zip › animals-2019181-supplementary-done.pdf]

animals

MDPI

Supplementary Materials

Table S1. External, internal and total biosecurity scores based on their specific sub-categories on four commercial fattening pigs` farms.

	Fattening pigs` farms			
	**Farm 1**	**Farm 2**	**Farm 3**	**Farm 4**
External biosecurity subcategories (%)				
A. Purchase of breeding pigs, piglets and semen	100	100	96	96
B. Transport of animals, removal of carcasses and manure	38	48	48	38
C. Feed, water and equipment supply	33	33	40	13
D. Visitors and farmworkers	59	47	47	29
E. Vermin and bird control	50	50	50	20
F. Location of the farm	100	80	100	80
**External biosecurity**	**64**	**62**	**64**	**50**
Internal biosecurity subcategories (%)				
G. Disease management	20	80	20	20
H. Farrowing and suckling period	N/A	N/A	N/A	N/A
I. Nursery unit	86	86	27	21
J. Finishing unit	93	79	50	47
K. Measures between compartments, working lines and use of equipment	29	18	18	14
L. Cleaning and disinfection	60	50	40	10
**Internal biosecurity**	**55**	**54**	**31**	**20**
**Total biosecurity**	**60**	**58**	**48**	**35**

Table S2. Transport variables (loading time, transport duration, slipping, vocalization).

Variable	Fattening pigs` farms			
	**Farm 1**	**Farm 2**	**Farm 3**	**Farm 4**
Loading time (min)	45-55	37-45	50-55	31-35
Transport duration (min)	5-7	26-30	60-70	55-65
Slipping (%)	20	10	50	28
Vocalization (%)	40	3	18	5

Table S3. Multivariate regression models predicting carcass and meat quality traits.

	R Square	β	95% CI	*p* value	*p* value (model)
Carcass weight (kg)					
Farm TBS		-0.130	(-0.275, 0.015)	0.079	
Lairage time	0.086	0.112	(-0.031, 0.256)	0.123	0.039
Farm TBS×L		-0.013	(-0.027, 0.002)	0.081	
Carcass yield (%)					
Farm TBS		-0.052	(-0.128, 0.023)	0.174	
Lairage time	0.312	0.223	(0.148, 0.298)	<0.0001	<0.0001
Farm TBS×L		0.028	(-0.016, -0.001)	0.028	
Chilling yield (%)					
Farm TBS		0.007	(0.005, 0.009)	<0.0001	
Lairage time	0.655	0.012	(0.010, 0.015)	<0.0001	<0.0001
Farm TBS×L		-6.556E-005	(0.0001, 0.0001)	0.549	
Temperature 24h (°C)					
Farm TBS	0.126	-0.021	(-0.035, -0.007)	0.003	0.006

Lairage time		0.014	(0.0001, 0.028)	0.045	
Farm TBS×L		0.0001	(-0.002, 0.001)	0.710	
a* value					
Farm TBS		0.049	(0.001, 0.097)	0.046	
Lairage time	0.136	0.057	(0.010, 0.105)	0.019	0.004
Farm TBS×L		0.005	(0.0001, 0.010)	0.032	

CI: confidence interval; TBS: total biosecurity score, L: lairage time, Farm TBS×L: interaction
